# Public perceptions of genetic sequencing in China: barriers and drivers of adoption

**DOI:** 10.1038/s41431-026-02109-7

**Published:** 2026-04-27

**Authors:** Liyong Lu, Shan Jiang, Yuan Wang, Sidong Liu, Jiao Lu, Yuanyuan Gu

**Affiliations:** 1 West China School of Public Health and West China Fourth Hospital, Sichuan University, Chengdu, China; 2 Macquarie University Centre for the Health Economy, Macquarie Business School & Australian Institute of Health Innovation, Macquarie University, Sydney, NSW Australia; 3 School of Management, Shanxi Medical University, Jinzhong, China; 4 Centre for Health Informatics, Australian Institute of Health Innovation, Macquarie University, Sydney, NSW Australia; 5 School of Public Policy and Administration, Xi’an Jiaotong University, Xi’An, China

**Keywords:** Population screening, Health policy, Genetic testing

## Abstract

This study explores public attitudes toward genetic sequencing (GS) services in China and identifies key factors influencing adoption. Although GS has the potential to strengthen precision public health, its uptake may be constrained by societal and attitudinal barriers. We used a sequential mixed-methods design, combining focus group discussions with a structured ranking questionnaire. A total of 28 participants (mean age 48.9 years) were included. The qualitative analysis identified five major themes reflecting participants’ understanding, concerns, and expectations regarding GS. Quantitatively, the most influential factors for adoption were the characteristics of genomic information and financial accessibility. Demographic differences were observed: men prioritized cost, whereas women emphasized clarity of information; older adults focused on affordability, whereas younger participants valued transparency and the utility of information. Psychological concerns also emerged as an important barrier, including fears of genetic discrimination and emotional distress arising from the implications of genetic information. In contrast to European studies that often highlight privacy and data protection, participants in this study placed greater emphasis on informational and financial considerations. Despite the modest sample, thematic saturation was reached and the mixed-methods approach provides complementary qualitative and quantitative evidence. Addressing public concerns through transparent communication, education, psychological support, and affordability-focused policies will be important to facilitate integration of GS into healthcare systems. These findings provide culturally grounded evidence to inform precision public health in China and similar contexts.

## Introduction

Precision public health represents a transformative paradigm within modern health care, aiming to improve health outcomes and reduce health disparities by leveraging individual-level data, including genomic, behavioral, and environmental factors [1, 2]. A central premise is that interventions can be tailored more effectively when informed by a comprehensive understanding of an individual’s genetic profile. As a cornerstone of precision public health initiatives, genetic sequencing (GS) (in this paper, “genetic sequencing” refers specifically to health-related genomic sequencing - i.e., sequencing undertaken for clinical or public health purposes, including diagnostic sequencing (to identify genetic causes of suspected disease) and predictive/preventive sequencing (to assess future disease risk and inform prevention or surveillance). We do not include non-medical sequencing uses such as ancestry testing, direct-to-consumer recreational reports, forensic sequencing, or agricultural/industrial sequencing) has emerged as a powerful tool with the potential to reshape the way diseases are understood, predicted, and treated [3]. By uncovering genetic underpinnings of disease, GS offers opportunities to predict individual health risks, identify genetic predispositions to various conditions, and personalize treatment strategies to improve outcomes [4].

The potential of GS to transform clinical practice and public health systems is particularly significant as advances in genomic technologies continue to reduce the cost and complexity of sequencing [5]. These developments have facilitated broader access to genomic data, making it increasingly feasible to integrate GS into clinical and public health practice. However, widespread adoption of GS in public health settings faces several barriers. While technical challenges are well documented, comparatively less attention has been paid to the societal dimensions that influence uptake [6, 7]. Public perception, shaped by awareness, trust, and acceptance of new technologies, is likely to play a central role.

Despite its promise, public understanding of GS remains limited, and ethical, legal, and social concerns, including data privacy, informed consent, and genetic discrimination, may impede adoption [8, 9]. For GS to be successfully integrated into health care systems, it is essential to foster public trust and participation. Addressing concerns about potential misuse of genetic data and the societal implications of revealing genetic information, particularly with respect to insurance, employment, and family dynamics, is critical to enhancing public confidence [10, 11]. Moreover, ensuring that the public perceives clear benefits from GS, especially in disease prevention and personalized medicine, is important for increasing acceptance [12].

Although numerous studies have examined public attitudes toward GS in high-income countries, less attention has been given to low- and middle-income countries such as China, where cultural, socioeconomic, and health-system dynamics may differ substantially. These differences are likely to shape public views. For example, family-centered decision-making in China may heighten concerns about implications for relatives, including stigma and reproductive decisions, rather than focusing solely on individual autonomy [13]. Socioeconomic factors, including urban–rural income gaps, persistent out-of-pocket costs for specialty services, and variable insurance coverage, can make affordability a primary concern for many individuals [14]. Health-system features, such as uneven distribution of specialty services, shortages of trained genetic counselors and clinical genetics expertise, rapidly expanding national genomic initiatives, and evolving rules on genetic data governance, may affect both access to and trust in genomic services. Together, these cultural, socioeconomic, and system-level dynamics may lead to distinct priorities and concerns compared with findings from many high-income countries. This study addresses this gap by exploring public perceptions of GS services in China, focusing on barriers and facilitators to adoption.

Building on prior research conducted in Europe and other regions, we collected qualitative data through focus group discussions and quantitative data using a structured ranking questionnaire. Qualitative data were analyzed thematically, while the ranking data were analyzed using weighted ranking scores (WRS) to compare the relative importance of domains and attribute types. This approach captures both in-depth, contextualized perceptions and broader, quantifiable patterns. Ultimately, the goal of this study is to provide evidence-based recommendations for promoting the integration of GS into healthcare systems to advance precision public health initiatives in China and similar contexts. For instance, such integration can be leveraged for chronic disease risk prediction, targeted precision intervention, and the prevention and control of newborn birth defects within medical and health facilities.

## Materials and methods

### Study design

This study used a sequential mixed-methods design (QUAL → QUAN) to assess public perceptions and preferences regarding GS services [15]. First, focus group discussions explored participants’ understanding, concerns, and expectations regarding GS. Immediately after each focus group (we acknowledge that the timing of the focus group discussions and the rankings could have led to some bias in the rankings. However, by ensuring that the focus group discussions occurred first, we aimed to capture participants’ initial opinions before they were asked to rank specific attributes of GS. Furthermore, the survey was framed as a personal, individual exercise, encouraging participants to base their rankings on their own views rather than being influenced by the group discussion), the same participants completed a structured ranking questionnaire to quantify the relative importance of predefined domains and attribute types influencing GS adoption. Findings from both components were integrated during interpretation to provide complementary insights into barriers and drivers of adoption.

### Focus groups

To explore the public’s understanding, concerns, and priorities regarding GS, we conducted four focus groups. Discussions followed a semi-structured protocol to ensure coverage of core topics while allowing flexibility to probe emerging themes.

Six domains were predefined based on prior research [16–26] and expert consultation: characteristics of genomic information, psychological impacts, impacts on family, rights, service process, and financial factors. Each domain included several attribute types. Experts reviewed the domains and attribute types to ensure contextual relevance and cultural appropriateness. Details are provided in Table 1, and the questionnaire structure is described in Supplementary Text S1.

**Table 1:** Domains and attribute types.

Domains	Attribute types	Explanation
Characteristics of genomic information	Disease risk	The risk of developing a health condition in the future.
	Actionability	The availability of interventions for the health condition indicated by the variant.
	Disease severity	Whether the health condition has severe consequences.
	Hereditary risk	The risk of inheriting disease genes or diseases to offspring.
	Test validity	Whether the laboratory can accurately detect variant genes and predict the relationship between these genes and disease risks.
	Side effects	This refers to the negative impacts brought about by the genetic testing technology itself (excluding the effects caused by the test results).
	Disease onset age	This refers to the age at which the disease is predicted to occur.
Impacts on recipients	Psychological harms	The genomic information causes psychological harms to the recipient.
	Information overload	There is so much genomic information that recipient is not able to process.
	Discrimination	Losing the eligibility to be covered by insurance or being discriminated against when interacting with others due to the carrier status of a pathogenic variant.
	Privacy	Personal privacy is compromised.
Impacts on family	Stress for relatives	The genomic information causes psychological harms to the relatives of the patient.
	Financial burden to family	The cost of GS and subsequent interventions can place a financial burden on your family.
	Guilt about disease risk	The parents may feel guilty about the disease risk inherited to their child.
Rights	Selective receipt	Whether the patient has the right to selectively receive genomic information.
	Receive all information	Whether the patient has the right to know all genomic information.
Service process	Professional	Whether the doctor’s service is professional, whether their operation is standard.
	Communication (Pre-GS & Post-GS)	Whether the physicians have communicated with you before GS, and explained to you the concept of GS services and possible risks. The approaches include fact-to-face meetings, virtual meetings, telephones, emails, and mails.
	Feedback time	How long will it take to wait for the results and whether they are timely.
	Targeted	Whether GS targets specific diseases.
	Psychological support	Whether physicians provide psychological support for patients during the communication of genomic results.
	Process location	The place where GS was done.
	Informed consent form	Whether the informed consent is complete and accurate.
	Physician attitude	The doctor’s attitude during sequencing, whether positive or negative.
Financial factors	Cost	The cost of GS and result interpretation.
	Insurance coverage	The impact of genetic sequencing results on the insurance coverage and reimbursement rates for the tested individual.

Participants received background information about GS, including its role in predicting disease risk and guiding personalized health care decisions. Focus groups were conducted in Mandarin Chinese and audio-recorded with participants’ consent. Recordings were transcribed verbatim. Transcripts were translated into English using a structured process: initial translation by a bilingual researcher, followed by review and quality checks by two bilingual authors with expertise in both languages.

Focus groups were conducted on September 5, 6, 19, and 20, 2020, lasted 30–90 min, and included 6-8 participants per session. The research team assessed thematic saturation iteratively and observed no new themes emerging after later sessions, suggesting saturation had been reached.

### Participant recruitment

Participants were recruited from the general public in Shanxi Province (Northern China) during September 2020. Prior personal experience with GS was not required; recruitment targeted adults without specialized knowledge of GS to capture broader public perspectives. All participants were urban residents of Shanxi Province; no rural residents were included. Recruitment procedures and inclusion criteria are provided in Supplementary Text S2.

### Data analysis

Qualitative data were analyzed using thematic analysis [27]. The completed consolidated criteria for reporting qualitative research (COREQ) checklist is included in Supplementary Table S1 [28]. Two researchers independently coded transcripts and resolved discrepancies through discussion until consensus was reached. Coding involved an initial open-coding stage followed by development and refinement of themes. A third researcher reviewed the coding framework and themes to enhance consistency and rigor. Data were managed and organized in Microsoft Excel to support systematic coding and theme development.

Quantitative questionnaire data were summarized using descriptive statistics for participant demographics and responses. Ranking analyses were conducted to assess the relative importance of the six domains and their corresponding attribute types using weighted ranking scores (WRS). Subgroup analyses by gender and age were conducted descriptively to explore differences in preferences and concerns (subgroup comparisons by age and gender were exploratory; given the sample size we did not fit formal multivariable interaction models, and quantitative subgroup results should be interpreted with caution). Data management and quantitative analyses were conducted in Microsoft Excel. Details of analytic procedures are provided in Supplementary Text S3.

## Results

### Descriptive characteristics of participants

As shown in Table 2, 28 participants (mean age 48.86 years, SD 14.80) took part in the study. The sample was approximately gender balanced and included a range of educational backgrounds; most participants were married. Detailed distributions for education level, marital status, and session attendance are provided in Table 2.

**Table 2:** The descriptive characteristics of participants.

Variables	Descriptive statistics
Age	48.86 (14.80)^a^
*<60 years*	18 (64.3)^b^
*Female*	10 (55.56)
*Male*	8 (44.44)
*≥60 years*	10 (35.7)
*Female*	5 (50.0)
*Male*	5 (50.0)
Gender	
*Female*	15 (53.57)
*Male*	13 (46.43)
Education	
*Primary school*	2 (7.14)
*Middle school*	5 (17.86)
*High school*	11 (39.29)
*Junior college and above*	10 (35.71)
Marriage status	
*Married*	24 (85.71)
*Unmarried*	4 (14.29)
Focus group date^c^	
*0*	8 (28.57)
*1*	6 (21.43)
*2*	8 (28.57)
*3*	6 (21.43)
Total	28

^a^Continuous variables are presented as mean (standard deviation).

^b^Discrete variables are presented as frequency and percentage (%).

^c^0, 1, 2, and 3 respectively represent September 5th, 6th, 19th, and 20th, 2020.

### Qualitative analysis: key themes

Thematic analysis identified five key themes: limited understanding of GS, desire for disease risk prediction and intervention, financial concerns and affordability, psychological impacts, and the importance of professional support. These themes provide insight into factors shaping public attitudes toward GS and its adoption. (While demographic details were not linked to specific quotations from the qualitative focus groups, the quantitative ranking data that follow help to illuminate how gender and age shaped participants’ views on genetic sequencing. These analyses complement the qualitative findings and provide a clearer picture of the role demographic factors play in influencing attitudes).

#### Limited understanding of GS

Many participants reported limited understanding of GS and its potential applications. One participant asked:

“*Is genetic testing just about going to the hospital to check for diseases and then treat them?*”

This suggests a gap in awareness and comprehension of GS services, highlighting the importance of public education.

#### Desire for disease risk prediction and intervention

Participants expressed strong interest in disease risk prediction and preventive interventions enabled by GS, particularly for chronic conditions such as hypertension and cancer. One participant noted:

“*I hope to know if I have a genetic predisposition to diseases like high blood pressure, so I can take preventive actions*”.

Another respondent noted:

“*Once I know the disease risks, I hope to consult professionals who can provide targeted medical assistance*”.

These perspectives aligned with the quantitative results, in which disease risk prediction ranked highly.

#### Financial concerns and affordability

Financial accessibility was a recurring concern, particularly regarding the cost of GS services (in China, patient-facing prices for genomic testing remain a substantial barrier to uptake. Market data compiled by China CDC Weekly indicate that consumer SNP-chip kits typically cost 199–799 CNY, whereas whole-genome sequencing kits are priced around 3999 CNY, reflecting the higher price of comprehensive sequencing [29]. Clinically, a nationwide survey of 20,132 physicians identified high price as the most common problem encountered when using genetic testing for rare-disease care, consistent with affordability concerns and uneven reimbursement [30]. Although the underlying technology costs of sequencing have fallen markedly over the past decade, out-of-pocket expenses for comprehensive tests remain non-trivial for many patients). One male participant stated:

“*Cost is the most important factor, without money, you can’t do anything*”.

Another one participant suggested:

“*Will GS be affordable for ordinary people? Can medical insurance cover the costs?*”

Older participants expressed heightened concern about affordability. Consistent with the ranking results, financial factors were among the most important determinants of adoption, particularly among participants aged 60 years and above.

#### Psychological impacts on recipients and their families

Participants raised concerns about the psychological consequences of receiving genetic information, particularly when results suggested risk of serious conditions with limited treatment options. One participant explained:

“*If one person in the family tests positive for a disease risk, it affects the mental well-being of everyone in the family. If I know the risk and the severity of the disease, especially for diseases with no good treatment options, it would put a huge psychological burden on the individual and the family*”.

Concerns about genetic discrimination were also raised, including implications for marriage and employment:

“*For unmarried individuals, if the test predicts future diseases, they may face discrimination, affecting their marriage prospects*”.

These findings indicate a need for counseling and psychological support to mitigate distress and support informed decision-making.

#### Service process and the role of professional support

Although logistical aspects (for example, scheduling and location) were not major concerns, participants emphasized the importance of professional support and clear communication throughout the process. One participant suggested:

“*For serious diseases, the test results should be communicated gently, with psychological support to help alleviate the emotional burden*”.

Another noted:

“*I want to hear the doctor explain the testing process before and after the test, and the results must be accurate and timely*”.

This aligns with the comparatively low ranking of service-process factors, while highlighting the value placed on communication and professional guidance.

### Quantitative analysis: ranking analysis for six domains

The weighted ranking analysis revealed systematic variations in the public’s prioritization of domains affecting the adoption of GS services. These domains were ranked according to their perceived importance in influencing the adoption process.

#### Overall priorities

**Fig. 1:**
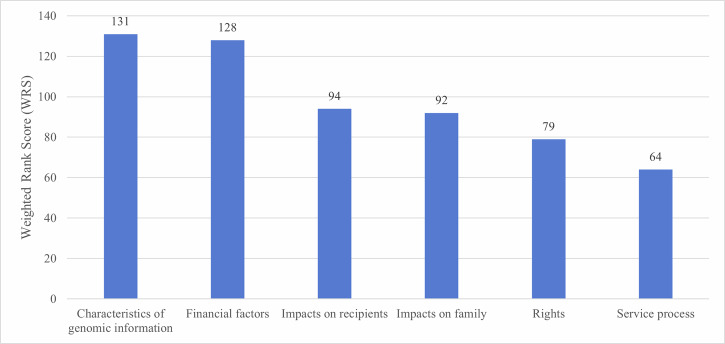
The results of ranking analysis for six domains based on weight score. The ranking is assigned the following weights: 6 points for the first position, 5 points for the second position, 4 points for the third position, 3 points for the fourth position, 2 points for the fifth position, and 1 point for the sixth position. The frequency of each ranking position for each domain is multiplied by the corresponding weight, and the sum is calculated to obtain the weighted score for that domain, which reflects the relative importance of the domain.

Across the entire sample (Fig. 1), the most highly prioritized domains were characteristics of genomic information (WRS = 131) and financial factors (WRS = 128). These were followed by impacts on recipients (WRS = 94), impacts on families (WRS = 92), rights (WRS = 79), and service process (WRS = 64).

#### Gender-based differentiation

**Fig. 2:**
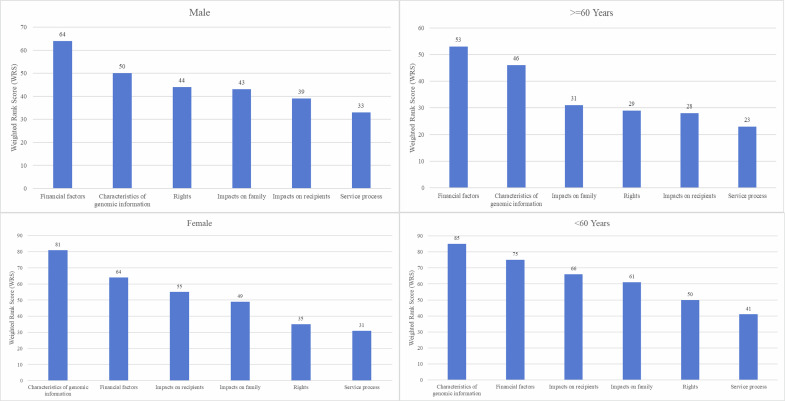
The results of ranking analysis for six domains based on weight score by genders and ages. The ranking is assigned the following weights: 6 points for the first position, 5 points for the second position, 4 points for the third position, 3 points for the fourth position, 2 points for the fifth position, and 1 point for the sixth position. The frequency of each ranking position for each domain is multiplied by the corresponding weight, and the sum is calculated to obtain the weighted score for that domain, which reflects the relative importance of the domain.

Gender-stratified results (left panel of Fig. 2) showed that men ranked financial factors highest (WRS = 64), followed by characteristics of genomic information (WRS = 50). Women ranked characteristics of genomic information highest (WRS = 81), followed by financial factors (WRS = 64). Both groups ranked service process lowest, suggesting that logistical aspects were not a major concern.

#### Age-stratified variations

Age-stratified results (right panel of Fig. 2) showed that participants aged 60 years and above (participants were categorized as <60 or ≥60 years of age, consistent with national demographic conventions and previous Chinese public-health studies [31], where 60 years marks the threshold for older adulthood) ranked financial factors highest (WRS = 53), whereas younger participants (<60 years) ranked characteristics of genomic information highest (WRS = 85). These patterns suggest that older adults prioritized affordability, while younger participants placed relatively greater emphasis on information clarity and utility.

### Quantitative analysis: ranking analysis for attribute types

Across the entire sample (Supplementary Fig. S1), the highest-ranked attribute type was disease risk (WRS = 33), followed by psychological harms (WRS = 22) and actionability (WRS = 21). Subgroup patterns by gender (Supplementary Fig. S2) and age (Supplementary Fig. S3) are described in Supplementary Text S4.

## Discussion

This study provides evidence on public perceptions of GS services in China and identifies key factors shaping adoption. Overall, characteristics of genomic information and financial considerations were most influential. Gender- and age-related differences further suggest that communication and policy responses may need to be tailored to different groups.

### Public priorities: genomic information and financial factors

Both qualitative and quantitative findings indicate that clarity and actionability of genomic information, together with financial accessibility, are primary drivers of GS adoption. This is consistent with studies highlighting the importance of interpretable and actionable results for integrating GS into health care [32]. Participants sought not only disease risk information but also guidance on personalized prevention and intervention, consistent with broader shifts toward precision medicine [1].

At the same time, cost emerged as a major barrier, particularly among older participants, echoing concerns about affordability and health care access [33]. Wider adoption may therefore require integration of GS into insurance schemes and/or targeted subsidies, especially for vulnerable groups. However, our data suggest that “financial factors” should not be interpreted as price sensitivity alone. Participants often evaluated affordability relative to household resources and, critically, in relation to whether GS should be treated as a consumer purchase or as a health service supported through reimbursement and broader public financing. In this context, out-of-pocket payment can signal not only economic burden but also uncertainty about legitimacy, value, and the appropriate locus of responsibility (individual versus health system), which may help explain why financial factors remained highly prioritized even when participants expressed strong interest in GS benefits.

The relative prominence of informational clarity and cost, and the lesser emphasis on privacy, likely reflects multiple contextual factors rather than a single explanation. First, trust in health systems and providers may shape which risks are perceived as most salient [34–36]. Second, family- and marriage-related implications may be experienced as immediate consequences of genetic information, elevating concerns about discrimination and psychological harms relative to more diffuse privacy concerns [37, 38]. Third, heterogeneity across Chinese subpopulations, including shifts in values alongside persistent family-oriented norms, may produce complex prioritization patterns [39, 40]. Together, these contextual factors suggest that policy and service design must address not only technical performance and pricing, but also the social meanings attached to genomic information and payment mechanisms.

### Psychological concerns and the role of professional support

Psychological impacts were a prominent barrier, especially when results suggested risk of severe or untreatable conditions. Participants also expressed concerns about genetic discrimination, particularly in employment and marriage. Prior research shows that genetic testing can induce distress, especially when results are uncertain or indicate predisposition to untreatable conditions [41, 42]. Incorporating counseling into GS services may mitigate these harms. Pre- and post-test counseling and empathetic communication may support informed decision-making and improve acceptance.

Our findings further indicate that psychosocial risks were often framed as extending beyond the individual to the family unit. Participants described anxiety, stigma, and discrimination as socially consequential, including concerns about marriage prospects and broader family reputation. In a family-centered decision environment, genetic risk information may be perceived as affecting not only one person’s opportunities but also the standing of the family, which can amplify perceived psychological burden and heighten the perceived costs of learning risk information. This framing offers a plausible explanation for why psychological harms ranked highly and why “professional support” and careful communication were repeatedly emphasized by participants (e.g., gentle delivery of serious results and psychological support during result disclosure). These findings align with prior work describing how stigma, metaphor, and culturally embedded communication dilemmas can shape responses to hereditary risk information in Confucian-influenced contexts [37, 38].

### Gender and age differences in preferences

Men tended to prioritize financial factors, whereas women emphasized clarity and transparency of genomic information. These differences may reflect gendered roles and differences in engagement with health information, suggesting value in gender-sensitive communication strategies [43, 44]. Older adults were particularly concerned about affordability, whereas younger participants placed relatively greater emphasis on information utility and predictive value. Educational messaging could therefore be tailored by group.

In practice, this suggests that for older adults, communication and service design may need to foreground affordability solutions (e.g., clear pricing, reimbursement pathways, and guidance on insurance coverage), whereas for younger adults, emphasis on validity, interpretability, and actionable follow-up may be particularly salient. For men, affordability and payment mechanisms may be especially influential, while for women, clarity of information and transparent explanation of benefits and limitations may be more central.

### Sample size and the methodological rigor

The sample size of this study (*n* = 28) is modest but consistent with similar qualitative and mixed-methods studies that explore public attitudes toward genetic testing. Previous studies of this nature typically involve focus groups with 15–30 participants to extract in-depth, qualitative insights [45]. While this sample size may not fully represent the broader population, it is adequate for capturing the nuanced views of participants and achieving thematic saturation, as no new themes emerged after the initial groups. This is consistent with guidance from [46], which suggests that thematic saturation is often reached within 4–8 focus groups or 9–17 participants. Moreover, the sequential mixed-methods design provided complementary qualitative and quantitative evidence, enabling us to connect contextualized perceptions with structured prioritization patterns.

### The comparison with previous studies in other regions

In comparison with studies conducted in Europe, our findings on public attitudes towards GS in China reveal both similarities and distinct regional differences. For example, Henneman et al. [47], Rosso et al. [48], and Haga et al. [49] all emphasize concerns about cost and affordability as significant barriers to GS adoption, a theme also present in our study. However, while our participants focused on financial accessibility, particularly among older individuals, European studies often highlight issues of equity and insurance coverage, reflecting differences in healthcare financing systems. The prominence of financial concerns in our study aligns with the socio-economic challenges in China, while European studies place greater emphasis on the role of public health systems and regulatory frameworks to address these concerns.

When it comes to privacy and data-sharing concerns, European studies such as Henneman et al. [47] and Haga et al. [49] identify these issues as central barriers to adoption, often tied to concerns about genetic discrimination in employment and insurance. In contrast, while privacy was mentioned in our study, it was less of a focus compared to the clarity of information and the financial accessibility of GS services. One plausible interpretation is that privacy concerns may be weighed against perceived utility and institutional trust, such that expectations regarding governance and stewardship can influence how privacy risks are perceived and prioritized in this context [50]; this does not imply that privacy is unimportant, but that it may be articulated through a different balance of priorities than in European settings where data protection frameworks and public discourse on privacy are highly salient. Furthermore, both our study and the European research agree on the importance of actionability and clarity of genetic information. Participants across all regions prioritized the need for genetic testing results to be clear, actionable, and linked to personalized health interventions, reflecting a universal expectation for GS to contribute directly to health outcomes.

Lastly, psychological impacts and the potential for social stigma emerged as significant concerns in both our study and European studies, though the focus of these concerns differed. In China, concerns were largely centered around family implications, such as marriage prospects, whereas European studies often emphasized concerns around employment and insurance discrimination. These regional differences likely reflect varying cultural norms and societal concerns. For instance, in China, family dynamics and social consequences are more immediately tangible, whereas European contexts tend to frame genetic testing within broader social and legal debates about individual rights and data protection.

### Implications for public health policy

Improving affordability is critical for equitable access, particularly for older adults and individuals with lower socioeconomic status. Integrating GS into public insurance schemes or providing subsidies could reduce barriers. In addition to lowering absolute prices, policy approaches that clarify reimbursement pathways and reduce out-of-pocket uncertainty may be especially influential, given that participants often interpreted affordability through expectations about insurance coverage and public financing responsibilities.

Public education campaigns can improve genomic literacy, address misconceptions, and explain benefits and limitations of GS. Communication should emphasize interpretability and actionability (i.e., what the results mean and what can be done), while also preparing individuals for potential psychosocial impacts. Given the salience of family-linked stigma and discrimination concerns, services should embed counseling and psychologically supportive result disclosure, and clearly communicate protections and governance arrangements for genetic data.

Insights from the Chinese experience may be useful for comparison with efforts to implement genomic medicine in Europe and other regions. While themes such as access, privacy, and the need for effective communication appear broadly shared, their relative salience and interpretation are shaped by local cultural values and policy frameworks. For example, the financial concerns identified here relate primarily to affordability and out-of-pocket uncertainty rather than equity debates within established coverage models, and privacy concerns were often expressed through the lens of clarity, trust, and appropriate use rather than data protection alone. Finally, the capacity for strong institutional coordination to rapidly scale new health technologies in China may contrast with more decentralized implementation pathways elsewhere, underscoring that similar goals (e.g., equitable access and trustworthy governance) may require different levers in different systems.

### Limitations and future research

Several limitations should be acknowledged. As a qualitative study, our findings are intended to provide an in-depth, contextualized understanding rather than statistical generalizability. Participants were urban residents recruited from a single province; therefore, the results should be interpreted as reflecting urban perspectives within this setting. Future studies should purposively include rural populations and additional regions to explore how social context, healthcare access, and local norms may shape perceptions of genetic sequencing services. Subgroup comparisons were descriptive and exploratory without inferential statistical testing. Because data were collected in 2020, perceptions may have been influenced by the early COVID-19 context. Finally, as in all qualitative research, interpretive subjectivity cannot be fully eliminated despite steps taken to enhance rigor. Future studies could include more diverse populations (including rural residents), health care providers, and patients. Preference-based methods such as discrete choice experiments (DCEs) could quantify trade-offs among attributes (for example, affordability, disease risk prediction, and actionability).

## Conclusion

This study contributes evidence on public perceptions of GS services in China and identifies key factors shaping adoption. Characteristics of genomic information and financial accessibility were the most influential domains, with psychological concerns and fears of discrimination also acting as barriers. The findings support policies that improve affordability, strengthen transparent communication and education, and embed counseling and professional support within GS services. With continued research and appropriate policy development, GS could support China’s precision public health strategy by enabling earlier detection, guiding population-level prevention programs, and improving equitable access to personalized health care.

## Supplementary information

Supplementary file

## Data Availability

Due to ethical and privacy considerations, the data generated or analyzed during this study are not publicly available. However, data can be made available upon reasonable request from the corresponding author.
