# Supplementary material for: Public perceptions of genetic sequencing in China: barriers and drivers of adoption
Source: Eur J Hum Genet. 2026 Apr 27;34(6):852–60. doi: 10.1038/s41431-026-02109-7 (PMC13247076; doi:10.1038/s41431-026-02109-7)
Supplement: Supplementary file 1 — Supplementary file [file 41431_2026_2109_MOESM1_ESM.docx]

**Supplementary file**

**Public Perceptions of Genetic Sequencing in China: Barriers and Drivers of Adoption**

**Content**

[**Text S1** The questionnaire structure 2](#_Toc216509543)

[**Text S2** The details of recruitment and the inclusion criteria 3](#_Toc216509544)

[**Text S3** The details of data analysis 4](#_Toc216509545)

[**Text S4** Quantitative analysis: Ranking analysis for attribute types 6](#_Toc216509546)

[**Figure S1** The results of ranking analysis for top 3 attribute types based on weight score 7](#_Toc216509547)

[**Figure S2** The results of ranking analysis for top 3 attribute types based on weight score by genders 8](#_Toc216509548)

[**Figure S3** The results of ranking analysis for top 3 attribute types based on weight score by ages 9](#_Toc216509549)

[**Table S1** Completed COREQ checklist 10](#_Toc216509550)

# **Text S1** The questionnaire structure

The questionnaire was designed to include three sections. (1) Demographic and socioeconomic information: participants provided information on age, gender, education level, and marital status. (2) Public discussion on GS: this section sought participants’ open-ended responses to a range of questions about their understanding of GS, concerns, and perceived benefits. This qualitative data was used to identify key themes in qualitative analysis. (3) Ranking of GS domains: participants were asked to rank the six domains and their corresponding attribute types in terms of importance, with a particular focus on identifying what they believed would drive or hinder the adoption of GS.

# **Text S2** The details of recruitment and the inclusion criteria

Recruitment efforts were carried out through multiple channels, including community outreach in hospitals, local community centers, and social media networks, to ensure broad representation across different social groups.

Inclusion criteria were: adults aged 18 years and older; no prior specialized knowledge or experience with genetic sequencing; a balanced representation in terms of gender and age. Recruiting a diverse participant pool ensured that the findings were reflective of varied demographic and social contexts, enhancing the generalizability of the study to broader public health initiatives. A total of 28 respondents participated in the four focus groups.

# **Text S3** The details of data analysis

Qualitative analysis: The qualitative data obtained from the focus group discussions were analyzed using thematic analysis. This approach enabled the identification of recurring themes and patterns in participants’ responses.

Quantitative analysis: Quantitative data from the questionnaire were analyzed using descriptive statistics to summarize participant demographics and responses. A ranking analysis was conducted to determine the relative importance of the six domains and their corresponding attribute types. Each factor was assigned a weight based on its rank, using a scoring system where the most important factor received the highest score. The weighted scores were then summed to obtain an overall ranking of each factor.

Specifically, referring previous study, the ranking analysis process as follows (Wang et al., 2023). For six domains, the ranking is assigned the following weights: 6 points for the first position, 5 points for the second position, 4 points for the third position, 3 points for the fourth position, 2 points for the fifth position, and 1 point for the sixth position. The frequency of each ranking position for each domain is multiplied by the corresponding weight, and the sum is calculated to obtain the weighted score for that domain, which reflects the relative importance of the domain. For attribute types, during the focus group process, given that the questionnaire involves as many as 26 attribute types, asking the interviewees to rank all 26 attribute types would be extremely difficult for patients to understand and operate, making it harder for them to identify the most important and concerning attribute type. Based on this, the researchers adopted a two-step approach: first, they asked the participants to identify the three most important attribute types from the 26, and then rank only these three attribute types. The ranking is assigned the following weights: 3 points for the first position, 2 points for the second position, and 1 point for the third position. The frequency of each ranking position for each attribute is multiplied by the corresponding weight, and the sum is calculated to obtain the weighted score for that attribute, which reflects the relative importance of the attribute.

Subgroup analyses were also conducted based on gender and age to explore differences in preferences and concerns. These analyses allowed for a deeper understanding of how demographic variables influence public perceptions of GS. The management and analysis of data are carried out using software of Microsoft Excel.

# **Text S4** Quantitative analysis: Ranking analysis for attribute types

**(1) Gender-based differentiation**

Subgroup analysis by gender revealed distinct preferences in how attribute types were prioritized (**Figure S2**). For male participants, the highest-ranked attribute type was psychological harms (WRS = 12), followed by cost (WRS = 11) and actionability (WRS = 11), and disease risk (WRS =9). This suggests that males were particularly concerned about the emotional burden of receiving genetic information, alongside practical financial considerations. Female participants, on the other hand, ranked disease risk (WRS = 24) as the most important attribute type, followed by cost (WRS = 17). Both actionability and psychological harms (WRS = 10) were equally ranked third. The higher ranking of disease risk for females indicates a greater concern for understanding genetic predispositions to health conditions compared to males.

**(2) Age-stratified variations**

Age-related differences in attribute preferences were also observed (**Figure S3**). For participants aged 60 years and older, the top-ranked attribute type was disease risk (WRS = 18), followed by actionability (WRS = 12) and psychological harms (WRS = 4). This suggests that older individuals are most concerned with understanding their disease risks and taking appropriate actions based on that information, with less emphasis on the psychological impact. Younger participants (<60 years) prioritized psychological harms (WRS = 18), followed by hereditary risk (WRS = 15) and cost (WRS = 10). These results indicate that younger participants are more concerned with the emotional consequences of genetic testing and the potential impact on their families, as well as financial considerations.

|  |
| --- |

**Figure S1** The results of ranking analysis for top 3 attribute types based on weight score

Notes: (1) During the focus group process, given that the questionnaire involves as many as 26 attribute types, asking the interviewees to rank all 26 attribute types would be extremely difficult for patients to understand and operate, making it harder for them to identify the most important and concerning attribute types. Based on this, the researchers adopted a two-step approach: first, they asked the participants to identify the three most important attribute types from the 26, and then rank only these three attribute types. (2) The ranking is assigned the following weights: 3 points for the first position, 2 points for the second position, and 1 point for the third position. The frequency of each ranking position for each attribute type is multiplied by the corresponding weight, and the sum is calculated to obtain the weighted score for that attribute type, which reflects the relative importance of the attribute type.

|  |
| --- |
|  |

**Figure S2** The results of ranking analysis for top 3 attribute types based on weight score by genders

Notes: (1) In the image of male, attribute type of “cost” and “actionability” are tied for second place; in the image of female, attribute type of “actionability” and “psychological harms” are tied for third place.

|  |
| --- |
|  |

**Figure S3** The results of ranking analysis for top 3 attribute types based on weight score by ages

# **Table S1** Completed COREQ checklist

| **COREQ item** | **Guide question/description** | **Answer** |
| --- | --- | --- |
| 1. Interviewer/facilitator | Which author/s conducted the interview or focus group? | The third author was the main facilitator. The second and fourth author acted as the assistants and instructors. |
| 2. Credentials | What were the researcher’s credentials? E.g. PhD, MD | The third author is MD, and the other authors have a PhD as their highest academic qualification. |
| 3. Occupation | What was their occupation at the time of the study? | Except for the third author who is a master’s student in the university, all other authors are researchers in university (including one professor, two associate professor, and one postdoctoral researcher, as well as one research fellow). |
| 4. Gender | Was the researcher male or female? | Two females and four males. |
| 5. Experience and training | What experience or training did the researcher have? | The first and fifth authors have extensive experience in health policy research and health economics research which have involved quantitative analyses. The second and last authors have extensive experience in preference elicitation studies which have involved questionnaire design, focus group design, and qualitative analyses. The third and fourth authors have extensive experience in quantitative analysis and the conducting of focus group, as well as survey. |
| 6. Relationship established | Was a relationship established prior to study commencement? | Yes: all co-authors have on-going research collaborations and have successfully collaborated on several research project. |
| 7. Participant knowledge of the interviewer | What did the participants know about the researcher? e.g. personal goals, reasons for doing the research. | At the beginning of the focus groups, the third authors introduced herself and the team including brief information about their respective positions as health economics researchers at university and their personal hobbies and interests. The main objectives of the research project were also explained. |
| 8. Interviewer characteristics | What characteristics were reported about the interviewer/facilitator? e.g. Bias. | See previous response. The main objectives of the research project were explained at the outset. |
| 9. Methodological orientation and theory | What methodological orientation was stated to underpin the study? e.g. grounded theory, discourse analysis, ethnography, phenomenology, content analysis. | Thematic analysis – key themes were identified inductively from the transcripts. |
| 10. Sampling | How were participants selected? e.g. purposive, convenience, consecutive, snowball. | A convenience sample was utilized based upon the key inclusion criteria (the details displayed in the Participant recruitment in the manuscript). Focus group sessions lasted between 30 to 90 minutes each and were audio-recorded with the participants’ consent. Each session consisted of 6-8 participants to maintain a manageable group size, facilitating effective discussions. |
| 11. Method of approach | How were participants approached? E.g. face-to-face, telephone, mail, email. | Face-to-face. |
| 12. Sample size | How many participants were in the study? | 28 (4 Focus group sessions with a total of 6-8 participants in each Focus group) |
| 13. Non-participation | How many people refused to participate or dropped out? Reasons? | Due to our on-site recruitment method, there are some respondents who refuse to participate due to personal preferences and time constraints. |
| 14. Setting of data collection | Where was the data collected? e.g. home, clinic, workplace | The first focus group was conducted in the home of interviewer, while others in the communities. |
| 15. Presence of non-participants | Was anyone else present besides the participants and researchers? | No |
| 16. Description of sample | What are the important characteristics of the sample? e.g. demographic data, date. | Described in Table 2 of the manuscript. |
| 17. Interview guide | Were questions, prompts, guides provided by the authors? Was it pilot tested? | Questions, prompts and focus group guides were developed by consultation between all the authors. Although these elements were not specifically pilot tested, they were constantly reviewed and updated between sessions. They also drew on prior qualitative work the authors had been involved in. |
| 18. Repeat interviews | Were repeat interviews carried out? If yes, how many? | No, we only ran focus groups. |
| 19. Audio/visual recording | Did the research use audio or visual recording to collect the data? | The research used audio recording. |
| 20. Field notes | Were field notes made during and/or after the interview or focus group? | Field notes were made during the focus group. |
| 21. Duration | What was the duration of the interviews or focus group? | Focus group sessions lasted between 30 to 90 minutes each. |
